# One-Pot Lipase-Catalyzed Enantioselective Synthesis of (*R*)-(−)-*N*-Benzyl-3-(benzylamino)butanamide: The Effect of Solvent Polarity on Enantioselectivity

**DOI:** 10.3390/molecules22122189

**Published:** 2017-12-09

**Authors:** Marina A. Ortega-Rojas, José Domingo Rivera-Ramírez, C. Gabriela Ávila-Ortiz, Eusebio Juaristi, Fernando González-Muñoz, Edmundo Castillo, Jaime Escalante

**Affiliations:** 1 The Center for Chemical Research, Autonomous University of Morelos State, Avenida Universidad 1001, Chamilpa, Cuernavaca 62210, Mexico; marina.ortega@uaem.mx (M.A.O.-R.); aghomes315@hotmail.com (J.D.R.-R); 2 Departamento de Química, Centro de Investigación y de Estudios Avanzados, Avenida I.P.N. 2508, Ciudad de México 07360, Mexico; gabriela@relaq.mx; 3 El Colegio Nacional, Luis Gonzáles Obregón 23, Centro Histórico, Ciudad de México 06020, Mexico; 4 Department of Cell Engineering and Biocatalysis, Institute of Biotechnology, UNAM, Apartado Postal 510-3, Cuernavaca C.P. 62271, Mexico; gonzalez@ibt.unam.mx

**Keywords:** lipase promiscuity, *aza*-Michael addition, stereoselectivity, enantioselectivity, chemoselectivity, solvent engineering

## Abstract

The use of the solvent engineering has been applied for controlling the resolution of lipase-catalyzed synthesis of β-aminoacids via Michael addition reactions. The strategy consisted of the thermodynamic control of products at equilibrium using the lipase CalB as a catalyst. The enzymatic chemo- and enantioselective synthesis of (*R*)-(−)-*N*-benzyl-3-(benzylamino)butanamide is reported, showing the influence of the solvent on the chemoselectivity of the *aza*-Michael addition and the subsequent kinetic resolution of the Michael adduct; both processes are catalyzed by CalB and both are influenced by the nature of the solvent medium. This approach allowed us to propose a novel one-pot strategy for the enzymatic synthesis of enantiomerically enriched β-aminoesters and β-aminoacids.

## 1. Introduction

The synthesis of β-amino acids and their derivatives is of great interest because these molecules have been shown to have a broad spectrum of biological properties [1,2,3]. In addition, β-amino acids are present in numerous natural products of complex molecules [1]. On the other hand, the incorporation of β-amino acids into β-peptides gives them structural characteristics capable of resisting hydrolytic degradation for long periods of time [4,5]. As we know, the particular configuration of biologically active β-amino acids is directly related to their biological activity and therefore to their therapeutic effect, so that the synthesis of these molecules with a certain absolute configuration has been an area of great interest. In this regard, many chemical strategies have been explored for the enantioselective production of β-amino acids. These methods involve, for example, chiral raw materials in the homologation of natural α-amino acids, the use of chiral auxiliaries in asymmetric synthesis, or the resolution of racemic mixtures of β-amino acids [1,3]. In this context, an alternative to conventional chemical methods for the resolution of racemic mixtures consists in the use of biocatalysts in non-aqueous media, a methodology that presents many advantages, including enzymes that show good to high catalytic activity in organic media [6,7,8]. However, the enzymatic resolution of racemic mixtures usually involves at least two steps, the synthesis of the racemic β-amino acid followed by enzyme-catalyzed resolution. Among the useful methodologies for the preparation of β-amino esters is the *aza*-Michael type addition of amines to α,β-unsaturated systems employing enzymes such as lipases [9,10,11,12].

On the other hand, several useful methodologies to control chemoselectivity in Michael addition have been developed, including the use of mutant enzymes and wild lipases, together with strategies such as solvent engineering [9,11,13]. Some authors have investigated different solvents for this type of reaction; however, the use of solvents has not always been intended as a resolutive process [9,10,11,12]. The kinetic resolution of racemic amino esters has usually been accomplished by enantioselective substitution reactions in acyl-substituted substrates [14], as well as via the resolution as a substrate for enzymatic *N*-acylation reactions [15,16]. Within this context, it has been described that nucleophilic substitution reactions in the acyl group can be achieved by hydrolysis [17], aminolysis [18], and transesterification [19], affording good yields and high enantioselectivity. In this regard, one of the most useful lipases is *Candida antarctica* lipase B (CalB), which is known for its catalytic promiscuity.

In 2009, our research group reported the successful use of solvent engineering strategies to control the chemoselectivity in the addition of benzylamine (**1**) to methyl crotonate (**2**) catalyzed by CalB [9]. In that report it was emphasized that the *aza*-Michael addition product (**4**) was preferably accumulated in a hydrophobic medium such as *n*-hexane. The desired β-amino ester **4** showed a specific rotation of [α]_D_^20^ = +0.45 (*c* 1.0, CHCl_3_), whereas the aminolysis product, crotonamide (**3**), was preferentially accumulated in polar solvents such as 2-methyl-2-butanol (2M2B), and a small amount of **4** obtained showed a specific rotation of [α]_D_^20^ = +11 (*c* 1.0, CHCl_3_). No other product was found. At that time, we did not comment on the origin of the observed enantiomeric excess (*ee*) and why it was achieved only in 2M2B. In that sense, if two reactions such as a nucleophilic addition and a substitution reaction were catalyzed by the same enzyme, one could anticipate that enantiopure esters could be obtained by a one-pot reaction, and that would imply the effect of the polarity of the solvent, not only for the control of the chemoselectivity but also in the enantioselectivity of the reaction, that is, in the resolution process. While the groups of Liese and Gröger reported that the origin of the enantiomeric excess does not result from an enantioselective Michael addition but by an enzymatic resolution via transamidation, their reactions were carried out in the absence of solvents and in a chemoenzymatic two-step process [18,20,21].

Therefore, in this work we carry out a one-pot reaction between benzylamine (**1**) and methyl crotonate (**2**) catalyzed by CalB to determine the effect of the polarity of the solvent, particularly its effect on the enantioselectivity of the enzymatic process.

## 2. Results and Discussions

### 2.1. Profile of Products Obtained in Solvents of Opposite Polarity: n-Hexane and 2M2B

**Scheme 1:**
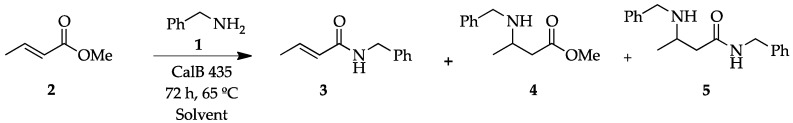
Lipase-catalyzed addition of benzylamine (**1**) to methyl crotonate (**2**) to yield aminolysis product (**3**), *aza*-Michael addition (**4**), and double addition product (**5**).

Several experiments were carried out to determine how chemo- and enantioselectivity are influenced by the polarity of the reaction medium in the addition reaction of benzylamine **1** to conjugated ester **2**. The experiments were carried out in triplicate in *n*-hexane and 2M2B (two solvents of opposite polarity). Furthermore, a control reaction was carried out in the absence of enzyme. Gas Chromatography-Mass Spectrometry (GC-MS) analysis of crude reaction products in the presence of CalB revealed the formation of the aminolysis product (**3**), the *aza*-Michael addition (**4**), and a double addition (**5**) (Scheme 1).

**Figure 1:**
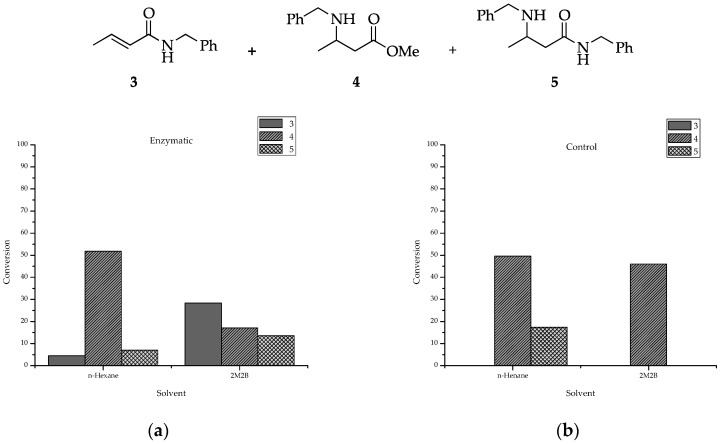
Influence of the polarity of the solvent in the chemoselective addition of **1** to **2**: (**a**) Lipase-Catalyzed addition; (**b**) In the absence of enzyme.

As shown in Figure 1a, the reaction medium determines the differentiated accumulation of the products. As anticipated, the enzyme is essential for the formation of crotonamide (**3**), since in the absence of enzyme, **3** was not detected (Figure 1b). Interestingly, in the presence of enzyme, **4** was preferentially accumulated in *n*-hexane, while in 2M2B the accumulation of **3** was favored. On the other hand, it was observed that **5** was formed in both media, although the proportion of **5** was slightly higher in 2M2B than in *n*-hexane. Finally, in the control reaction (Figure 1b), the *aza*-Michael adduct (**4**) was observed as the main product in the two media. It is noteworthy that the double-addition product (**5**) accumulated in *n*-hexane but not in 2M2B, so we can conclude that 2M2B is not a suitable solvent for the formation of **5** in the absence of enzyme.

### 2.2. Enantioselectivity

As discussed above, in addition to the formation of products **3** and **4**, the double addition product **5** was also detected. With *n*-hexane as the reaction medium **3**, **4**, and **5** were obtained in 7, 81, and 12% yield, respectively. It should be noted that both product **4** and **5** presented *ee* values which were determined by Chiral HPLC. Thus, β-amino ester **4** exhibited an *ee* of 1.1% {[α]_D_^20^ = +0.3 (*c* 1.0, CHCl_3_)} and the β-aminoamide **5** showed an *ee* of 3.4% {[α]_D_^20^ = − 1.4 (*c* 1.0, CHCl_3_)} (entry 1, Table 1). However, when the medium was 2M2B, the proportions of **3**, **4**, and **5** were 48%, 29%, and 23%, respectively. In contrast to the results obtained in *n*-hexane, the *aza*-Michael adduct **4** obtained in 2M2B showed an *ee* of 96% {[α]_D_^20^ = + 15.6 (*c* 1.0, CHCl_3_)}. Finally, the product **5** obtained in 2M2B showed an *ee* of 72% {[α]_D_^20^ = −19.2 (*c* 1.0, CHCl_3_)} (entry 3, Table 1). These results are consistent with those obtained in our previous report [9].

**Scheme 2:**
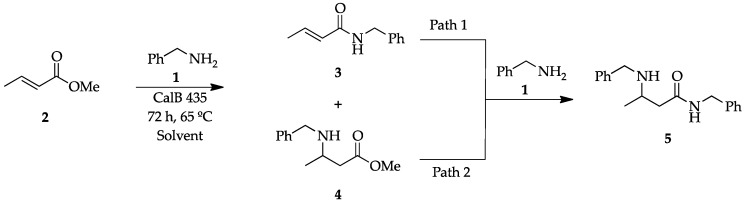
Possible routes for the formation of product **5** by the *aza*-Michael addition of **1** upon the substrate **3** (Path 1), or by the aminolysis process upon the adduct **4** (Path 2).

As shown in Scheme 2, compound **5** could be produced by the *aza*-Michael addition of benzylamine **1** on substrate **3** (Path 1) or by the nucleophilic substitution of the acyl group on substrate **4** (Path 2). To clarify which of the two routes is correct, the following experiments were carried out.

The first experiment consisted in placing compound **3** (0.5 M in 2M2B) in the presence of CalB (10 mg mL^−1^), 0.75 equiv. of benzylamine **1**, and 3Ǻ molecular sieves; this mixture was heated to 65 °C for 72 h. The enzyme was removed by filtration and washed with dichloromethane (DCM) and the reaction crude was analyzed by thin layer chromatography (TLC) and ^1^H-NMR, noting that no formation of compound **5** took place.

In a second experiment and after synthesizing *rac*-**4** according to the literature [22], *rac*-**4** was placed in 2M2B (0.5 M) together with benzylamine **1** (0.75 eq), CalB (10 mg mL^−1^), and molecular sieves at 65 °C for 3 h. The crude product from the reaction was analyzed by TLC and ^1^H-NMR, observing the formation of compound **5** in 41%. The latter result confirms the origin of product **5** by nucleophilic substitution of the acyl group on substrate **4** (Path 2). After the isolation of compound **4**, it showed a specific rotation of [α]_D_^20^ = + 7.9 (*c* 1.0, CHCl_3_) and for compound **5** of [α]_D_^20^ = − 24.5 (*c* 1.0, CHCl_3_). This result is also in agreement with the observations made by Gröger and collaborators [18,20,21].

**Scheme 3:**
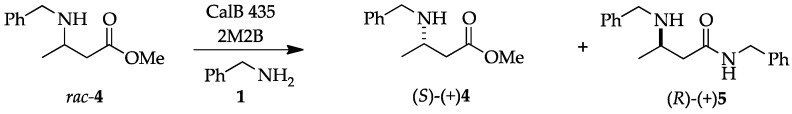
Resolution of *rac*-**4** in 2M2B with CalB.

The new results clarify the origin of the enantiomeric excess of the compound (*S*)-(+)-**4**, obtained in 2M2B, by a resolution process with CalB on the compound *rac*-**4** (Scheme 3).

To confirm whether the presence of 2M2B played a significant role in the resolution reaction, it was decided to repeat the reaction in a 1:1 mixture of *n*-hexane/2M2B. Under these conditions, the three products **3**, **4**, and **5** were again found in yields of 48%, 29%, and 23% respectively, and although the proportions did not change with respect to those obtained in 2M2B, the *ee* value of the Michael adduct and the double addition product decreased to 79.8% and 67.8% respectively (entry 5, Table 1). Additionally, it is important to mention that in the control reaction both the Michael adduct and the double addition product were found in a ratio of 90:10, respectively. These results confirm that **5** is only obtained by an enzymatic process in 2M2B, since in the control reaction with 2M2B this was not observed (entry 4, Table 1). Additionally the low *ee* of compound **5** obtained in *n*-hexane is probably due to the fact that the chemical process is much faster than the enzymatic process.

### 2.3. Optimization of Enantiomeric Excess

The best values of *ee* for (*S*)-(+)-**4** and (*R*)-(−)-**5** were obtained in 100% of 2M2B, but this solvent also favors the formation of aminolysis product **3**. Thus, it was decided to design a system in which the formation of **3** could be minimized in order to favor the process of resolution of *rac*-**4**. This new system was *n*-hexane/2M2B 90:10. The reaction was monitored by ^1^H-NMR and proportions were determined by GC-MS. The products **3**, **4**, and **5** were found in yields of 25%, 28%, and 47%, respectively, and isolated compound **4** showed an *ee* of 75% {[α]_D_^20^ = +7.7 (*c* 1.0, CHCl_3_)}. For compound **5**, the *ee* was 67% {[α]_D_^20^ = −21 (*c* 1.0, CHCl_3_)} (entry 7, Table 1). These results were anticipated in terms of the polarity of the solvent, and although the presence of *n*-hexane inhibited the formation of compound **3**, the formation of compound **5** was favored. Also, the optical rotation data were slightly smaller with respect to the reactions carried out in 50:50 of *n*-hexane: 2M2B.

## 3. Materials and Methods

*Candida antarctica* lipase B (CalB) was obtained as Novozym^®^ 435 (Novozymes Mexico, Mexico City, Mexico). Methyl crotonate **2** and benzylamine **1** were obtained from Sigma Aldrich (St. Louis, MI. USA). Thin layer chromatography (TLC) was carried out on pre-coated Merck silica gel 60 F_254_ (Merck Mexico, Mexico City, Mexico). Column chromatography was performed on Merck silica gel 60 (0.040–0.063 mm) (Merck Mexico, Mexico City, Mexico). Analytical grade solvents were purchased from Tecsiquim (*i*-PrOH) (Tecsiquim, Toluca, Mexico) or Caledon Laboratory Chemicals (*n*-hexane) (Georgetown, ON, Canada).

### 3.1. Analytical Methods

NMR spectra of products as well as the proportions of each product in the reaction mixture were recorded on Varian Gemini 200 MHz and Varian Mercury 400 MHz (^1^H-NMR), and 50 and 100 MHz (^13^C-NMR) spectrometers, using CDCl_3_ as a solvent and tretramethylsilane (TMS) as an internal standard. The proportions of each product in the reaction mixture were determine by Gas Chromatography-Mass Spectrometry on an Agilent GC system 6890 series coupled to an Agilent mass selective detector 5973N with an HP-5MS column length of 30 m, internal size of 0.25 mm, film thickness of 0.25 μm, split ration of 5:1, 40 °C min^−1^, 5 °C min^−1^ to 250 °C min^−1^, 10 °C min^−1^ to 280 °C min^−1^. Retention times (min): **4** = 25.3, **3** = 28, and **5** = 43. Enantiomeric excesses were measured by an HPLC Dionex Ultimate 3000 model and UV Shodex detector RI-101 model at 210 nm, using a Chiracel^®^ OD-H column (5 × 250 mm), hexane/*i*-PrOH 98/2 (*v*/*v*) as eluent at a flow rate of 0.8 mL min^−1^ at 25 °C for **4**. Retention times: (*R*)-**4**: 11 min, (*S*)-**4**: 13 min. A CHIRALPAK^®^ AS-H column (5 × 250 mm) was used for **5**, with hexane/*i*-PrOH 80/20 (*v*/*v)* as eluent at a flow rate of 1 mL min^−1^ at 25 °C. Retention times: (*S*)-**5**: 20 min, (*R*)-**5**: 29 min. Optical rotations were measured by means of a Perkin-Elmer 341 polarimeter.

### 3.2. Chemistry

#### 3.2.1. Enzymatic Reactions

The enzymatic reactions were carried out in sealed glass vials. The reaction consisted in the preparation of a solution of benzylamine (0.375 g, 3.5 mmol), methyl crotonate (0.525 g, 1.5 eq), CalB (10 mg mL^−1^), and 0.01 g of molecular sieves dissolved in 7 mL of a pure dry solvent or a solvent mixture. The reaction mixture was stirred in a thermostated water bath at 65 °C for 72 h. Enzyme was filtered off and washed with DCM and then methanol. The solvent was evaporated under reduced pressure. The proportions of **3**, **4**, and **5** in the reaction mixture were determined by GC-MS. The products were purified over SiO_2_ using AcOEt/DCM (98:02 to 60:40) and then AcOEt/MeOH (90:10 to 50:50), and their structures were confirmed by ^1^H and ^13^C-NMR, and compared with available information in the literature [21,22,23].

*N-Benzylcrotonamide*, (**3**): White solid, mp 114–116 °C. ^1^H-NMR (200 MHz, CDCl_3_) δ 1.83 (dd, *J* = 2 Hz, *J* = 8 Hz, 3H); 4.47 (d, *J* = 6 Hz, 2H); 5.82 (m, 1H); 5.96 (br s, 1H); 6.86 (m, 1H); 7.21–7.37 (m, 5H). ^13^C-NMR (100 MHz, CDCl_3_) δ 17.9, 43.7, 125.0, 127.6, 128.0, 128.8, 138.5, 140.4, 166.1. Spectral data ^1^H and ^13^C-NMR are consistent with the literature reports [23].

*(S)-(+)-Methyl 3-(benzylamino)butanoate*, [(*S*)-(+)-**4**]: Yellow oil, after isolation. [α]_D_^20^ = +0.3 (*c* 1.0, CHCl_3_), for product obtained in *n*-hexane; [α]_D_^20^ = +7.7 (*c* 1.0, CHCl_3_), for product obtained in *n*-hexane/2M2B, 9:1; [α]_D_^20^ = +11.6 (*c* 1.0, CHCl_3_), for product obtained in *n*-hexane/2M2B, 1:1; [α]_D_^20^ = +15.6 (*c* 1.0, CHCl_3_), for product obtained in 2M2B. ^1^H-NMR (200 MHz, CDCl_3_) δ 1.15 (d, *J* = 8 Hz, 3H); 1.75 (br s, 1H); 2.38 (dd, *J* = 16 Hz, *J* = 6 Hz, 1H); 2.51 (dd, *J* = 15 Hz, *J* = 6 Hz, 1H); 3.16 (m, 1H); 3.67 (s, 3H); 3.74 (d, *J* = 14, 1H); 3.84 (d, *J* = 12 1H); 7.19–7.41 (m, 5H). ^13^C-NMR (50 MHz, CDCl_3_) δ 20.6, 41.6, 49.9, 51.4, 51.7, 127.1, 128.3, 128.6, 140.5, 173.0. Spectral data ^1^H and ^13^C-NMR are consistent with the literature [22].

*(R)-(−)-N-Benzyl-3-(benzylamino)butanamide*, [(*R*)-(−)-**5**]: Yellow resin, after isolation. [α]_D_^20^ = −1.4 (c 1.0, CHCl_3_), for product obtained in *n*-hexane; [α]_D_^20^ = −21 (c 1.0, CHCl_3_), for product obtained in *n*-hexane/2M2B, 9:1; [α]_D_^20^ = −20.8 (c 1.0, CHCl_3_), for product obtained in *n*-hexane/2M2B, 1:1; [α]_D_^20^ = − 19.2 (c 1.0, CHCl_3_), for product obtained in 2M2B. ^1^H-NMR (200 MHz, CDCl_3_) δ 1.20 (d, *J* = 8 Hz, 3H); 1.82 (br s, 1H); 2.27 (dd, *J* = 16 Hz, *J* = 8 Hz, 1H); 2.46 (dd, *J* = 16 Hz, *J* = 4 Hz, 1H); 3.10 (m, 1H); 3.69 (d, *J* = 12, 1H); 3.80 (d, *J* = 14 1H); 4.42 (d, *J* = 6, 2H); 7.08–7.37 (m, 10H); 8.43 (br s, 1H). ^13^C-NMR (50 MHz, CDCl_3_) δ 20.3, 41.8, 43.5, 50.4, 51.0, 127.5, 127.5, 128.1, 128.5, 128.8, 128.9, 138.8, 171.8. Spectral data ^1^H are consistent with the literature [21].

#### 3.2.2. *Synthesis of rac-Methyl 3-(benzylamino)butanoate*, *rac*-**4** was Prepared According to the Literature

A solution of benzylamine (**1**, 0.10 g, 1 mmol) and methyl crotonate (**2**, 0.10 g, 1 mmol) in 3 mL of methanol were added to a 10-mL glass microwave reaction vessel containing a stir bar. The reaction vessel was sealed with a cap and then placed into the microwave cavity. The reaction was heated at T = 150 °C and 40 psi for 1 h. After the reaction was completed and the vessel was cooled to below 50 °C using a flow of compressed air, the solvent was evaporated in vacuo and the crude material was purified by column chromatography to give the product as a yellow oil. Yield: 62%. Spectral data ^1^H and ^13^C are consistent with those reported in the literature [22].

## 4. Conclusions

In this study, the origin of the *ee* for (*S*)-(+)-*4* obtained in 100% of 2M2B could be clarified as a resolution process on *rac*-**4**. First, the addition of benzylamine to methyl crotonate was catalyzed by CalB to obtain *rac*-**4**, followed by its resolution through an aminolysis reaction to obtain (*R*)-(−)-**5**. We found that the process in 100% of *n*-hexane is racemic, and also favors the chemical synthesis of **5**. In 2M2B, the synthesis of *rac*-**4** is unfavorable and favors the formation of compound **3**. Finally, we found a solvent engineering strategy to control the chemoselectivity in the addition of *aza*-Michael catalyzed by CalB and at the same time we demonstrated the direct resolution of an intermediate species through an aminolysis reaction.

## Figures and Tables

**Table 1:** Conversion and proportions of **3**, **4**, and **5** products obtained in the addition of benzylamine **1** to methyl crotonate **2** in *n*-hexane and 2M2B.

Entry	Enzyme	Solvent	Conversion ^a^	Proportion (%) ^b^ 3/4/5	1,4-Addition 4		Double Add 5	
					[α]_D_^c^	*ee* (%) ^d^	[α]_D_^c^	*ee* (%) ^e^
1	CalB 435	*n*-hexane, 100%	64	7/81/12	+0.3	1.1	-1.4	3.4
2	Control	*n*-hexane, 100%	67	ND/74/26	0	0	0	0
3	CalB 435	2M2B, 100%	59	48/29/23	+15.6	96	-19.2	72
4	Control	2M2B, 100%	46	ND/100/ND	0	0	0	0
5	CalB 435	*n*-hexane/2M2B, 50:50	67	48/29/23	+11.6	79.8	-20.8	67.8
6	Control	*n*-hexane/2M2B, 50:50	53	ND/90/10	0	0	0	0
7	CalB 435	*n*-hexane/2M2B, 90:10	84	25/28/47	+7.7	75	-21	67
8	Control	*n*-hexane/2M2B, 90:10	74	ND/80/20	0	0	0	0

Reaction conditions: 3.5 mmol of benzylamine **1**, 1.5 eq of methyl crotonate **2**, 70 mg of CalB (Novozyme 435), 0.1 g of molecular sieves, and 7 mL of solvent. Conversion was calculated in the basis of **1** consumption. ^a^ Yield determined as isolated product. ^b^ Determined as proportion by CG-MS. ^c^ (*c* 1.0, CHCl_3_). ^d^ *ee* determined by Chiral HPLC. Chiracel OD-H, hexane/*i*-PrOH 98:02, 0.8 mL min^−1^, UV-VIS 210 nm. Retention times: (*R*)-**4**: 11 min, (*S*)-**4**: 13 min. ^e^ *ee* determined by Chiral-HPLC. CHIRALPAK^®^ AS-H, hexane/*i*-PrOH 80:20. 1 mL min^−1^, UV-VIS 210 nm. Retention times (*S*)-**5**: 20.4 min, (*R*)-**5**: 29.4 min.
